# Volume targeted mask ventilation during simulated neonatal resuscitation – A randomized crossover manikin study

**DOI:** 10.1016/j.resplu.2025.101109

**Published:** 2025-09-23

**Authors:** Chelsea Morin, Kashmala Yousafzai, Brenda Hiu Yan Law, Georg M. Schmölzer

**Affiliations:** a Centre for the Studies of Asphyxia and Resuscitation, Neonatal Research Unit, Royal Alexandra Hospital, Edmonton, Alberta, Canada; b Department of Pediatrics, University of Alberta, Edmonton, Alberta, Canada

**Keywords:** Infants, Newborn, Neonatal resuscitation, Mask ventilation, Respiratory function tests

## Abstract

**Objective:**

To compare mask positive pressure ventilation (PPV) provided by pressure guided devices (i.e., T-Piece) with or without a respiratory function monitor (RFM) with ventilator-based volume-targeted ventilation (VTV) using a VN500 Draeger ventilator or the NextStep^TM^, a novel ventilation device designed for the delivery room.

**Methods:**

Prospective, randomized, crossover, simulation study. Following orientation to ventilation devices, participants were randomized to order of four ventilation devices (NextStep^TM^, VN500 Draeger ventilator, T-piece PPV with RFM visible, and T-piece PPV with RFM masked) and order of four simulation scenarios. The study was performed in a neonatal resuscitation room within a level 3 neonatal intensive care unit. Participants were trained neonatal resuscitation providers or instructors with experience as team leader. **Semi-automated, ventilator-based volume-targeted mask PPV (VTV-PPV) (NextStep^TM^ or Draeger Ventilator) was compared to manual PPV via a T-piece device (RFM either visible or masked).** Primary outcome was reduction in mask leak with the NextStep^TM^ compared to the other devices.

**Results:**

Thirty-two healthcare professionals [25 (78.1 %) were female and 7 (21.9 %) were male] participated. The median (interquartile range) mask leak was significantly lower with VTV-PPV with NextStep^TM^ [6 (1–12) %] compared to the Draeger Ventilator [24 (25–38)%, p = 0.01], T-Piece with RFM [18 (9–33) %, p = 0.0088], and T-Piece without RFM [32 (12–57)%, p = >0.0001]. The median (IQR) delivered tidal volume was not different between groups, although the NextStep^TM^ had less tidal volume variation compared to all other groups and peak inflation pressure was significantly lower with VTV-PPV with NextStep^TM^ compared to all other groups.

**Conclusion:**

In a neonatal manikin model, VTV-PPV with the NextStep^TM^ using a two-hand hold reduced mask leak compared to the T-piece without RFM guidance.

## Introduction

When newborn infants fail to initiate spontaneous breathing, the international resuscitation guidelines and various national resuscitation guidelines recommend positive pressure ventilation (PPV) as the cornerstone of respiratory support immediately after birth.^1^, ^2^, ^3^ The purpose of PPV is to create a functional residual capacity, deliver an adequate tidal volume (V_T_) to facilitate gas exchange, and stimulate breathing while minimizing lung and brain injury.^4^, ^5^ In the delivery room, PPV is routinely provided using a face mask connected to a pressure-limited device (called T-Piece resuscitator,^6^ where a peak inflation pressure is somewhat arbitrarily chosen with the assumption an adequate V_T_ will be delivered.^1^, ^2^, ^3^ However, the delivered V_T_ is rarely measured and therefore peak inflation pressure is not adjusted to optimize V_T_ delivery.^7^, ^8^ Furthermore, observational studies in the delivery room reported that 75 % of the time, healthcare professionals cannot accurately assess chest wall movement.^7^, ^8^ PPV is further complicated by an often widely varying leak between the mask and face and most healthcare professionals being unaware of the extent of their mask leak.^7^, ^9^, ^10^, ^11^, ^12^

A respiratory function monitor (RFM) has been postulated to reduce mask leak and improve V_T_ delivery.^13^, ^14^, ^15^, ^16^, ^17^, ^18^ Manikin studies reported that an RFM during training improves the effectiveness of mask ventilation.^13^, ^18^ Similarly, three randomized trials in the delivery room comparing an RFM either visible or masked during PPV reported that a RFM allows the healthcare professionals to adjust the V_T_ and remain more often within the target V_T_ range with lower rates of excessive (>8 mL/kg) V_T_ delivery.^14^, ^15^, ^17^, ^19^ While these studies confirm that having an RFM visible during mask PPV in the delivery room reduced high V_T_ delivery, this did not lead to an improvement in clinical outcomes.^16^, ^17^

While PPV in the delivery room is mainly pressure guided, in the neonatal intensive care unit ventilation strategies routinely use volume-targeted ventilation (VTV) with a set V_T_.^20^, ^21^ Meta-analyses of the use of volume-targeted ventilation in the neonatal intensive care unit showed a reduction in the combined outcome of death or bronchopulmonary dysplasia (typical RR [95 %CI] 0.73 [0.57–0.93] and the combined outcome of periventricular leukomalacia or grade 3–4 intraventricular hemorrhage (typical RR [95 %CI] 0.48 [0.28–0.84].^21^ Although PPV is commonly used in the delivery room, VTV is rarely used in this setting to provide respiratory support for infants during the first few minutes of their life.

Although the use of a ventilator in the delivery room may initially be perceived as overly complex and resource-intensive, a novel volume-controlled ventilator with an integrated respiratory function monitor, the NextStep^TM^ (KM Medical, Auckland, New Zealand), may offer a practical solution.^22^, ^23^, ^24^, ^25^ In randomized controlled manikin studies, the NextStep^TM^ provided the most consistent V_T_ compared to a T-Piece Resuscitator and self-inflating bag.^22^, ^23^ Similarly, during animal studies the NextStep^TM^ had the most consistent V_T_ delivery compared to T-Piece Resuscitator, self-inflating bag, flow-inflating bag, and a mechanical ventilator.^24^, ^25^ While previous simulation studies reported the feasibility of VTV using a routinely used mechanical ventilator,^26^, ^27^ no study has compared PPV provided by the NextStep^TM^ to PPV provided by T-Piece or mechanical ventilator. We aimed to compare pressure-controlled ventilation with or without an RFM with VTV during simulated mask ventilation on a neonatal manikin. We hypothesized that PPV guided by a NextStep^TM^ compared to PPV using either the Draeger Ventilator or the T-Piece Resuscitator with or without an RFM (i.e., the current standard of care) will reduce mask leak, to deliver more consistent V_T_, during simulated mask ventilation in a newborn manikin model.

## Methods

This was a prospective, randomized, crossover simulation study, carried out at the Royal Alexandra Hospital, Edmonton, a tertiary perinatal center admitting ∼150 infants <29 weeks’ gestation to the neonatal intensive care unit annually. The study was approved by the Human Research Ethics Board, University of Alberta (Pro00114108).

### Participants

Healthcare professionals working at the Royal Alexandra Hospital were recruited. They were eligible to participate if they were i) an Neonatal Resuscitation Program instructor and/or provider with certification, ii) experienced with mask ventilation in the delivery room, and iii) experienced with VTV in the neonatal intensive care unit.

### Outcomes

The primary outcome was reduction in mask leak with teh NextStep^TM^ compared to the other devices.

### Randomization

Participants were randomized to the order of PPV devices: “VTV-PPV with NextStep^TM^”, “VTV-PPV with Draeger Ventilator”, “T-Piece with RFM” or “T-Piece without RFM”. Each participant performed resuscitation maneuvers, including mask ventilation, for five minutes with each device. Randomization was done using variable block sizes. Randomization was done using https://www.randomizer.org, a web-based randomization program. Allocation concealment was achieved using sequentially numbered, sealed envelopes containing the allocation. These were opened just prior to the first simulation.

### Sample size calculation and power estimates

We hypothesized that the NextStep^TM^ would have the lowest mask leak. In previous simulation studies mean (SD) mask leak was 51 (20)% using a T-Piece without RFM.^26^, ^27^ A sample size of 32 was sufficient to detect a clinically important (20 %) relative reduction in mask leak from 51 % to 41 %, with 80 % power, and a 2-tailed α error of 0.05 for a randomized crossover study with four groups. Sample calculation was performed using Stata 17 (StataCorp LLC, College Station, TX).

### Blinding

Blinding was not possible during the simulation due to the nature of providing the various ventilation techniques. However, the statistical analysis was blinded to group allocation.

## Study protocol

### Ventilation devices

– Neopuff T-piece (Fisher & Paykel, Auckland, New Zealand), with default setting of peak inflation pressure of 24 cmH_2_O, positive end expiratory pressure of 6 cmH_2_O, flow rate of 10 L/min. Target ventilation rate (achieved by user providing PPV) of 40–60/min. A one-handed technique is used to provide PPV with the T-piece as a second hand is needed to cover and uncover the valve.
– NextStep^TM^ Neonatal Resuscitator (KM Medical, Auckland, New Zealand) with default settings of maximum pressure (Pmax) of 40 cmH_2_O, positive end expiratory pressure of 5 cmH_2_O, rate of 50/min, set V_T_ of 5 mL/kg, and inflation: expiration ratio of 1:3. The NextStep^TM^ delivers V_T_ with an accuracy of 0.1–0.3 mL (according to the manufacturer); it also controls ventilation rate and monitors airway pressure.^22^, ^23^, ^24^, ^25^ A two-handed technique is used during PPV.
– VN500 neonatal ventilator (Dräger Medical, Lübeck, Germany) with default settings of maximum pressure (Pmax) of 40 cmH_2_O, positive end expiratory pressure of 6 cmH_2_O, rate of 50/min, set V_T_ of 5 mL/kg. A two-handed technique is used during PPV.

None of the devices were connected to heated/humidified gas.

### Respiratory function monitors

A flow sensor was placed between the mask and the ventilation device to record V_T_ and airway pressures. With the T-Piece, a Monivent Neo Training (Monivent, Göteborg, Sweden) flow sensor was used, while a hot-wire anemometer was used for the VN500 Ventilator, and a digital flow sensor for the NextStep^TM^. During PPV, participants were asked to assess mask leak and V_T_ delivery on the Monivent Neo Training screen and the screens of the VN 500 Ventilator and the NextStep^TM^ and adjust their mask position to reduce mask leak.

### Simulation protocol

There were four groups: “VTV-PPV with NextStep^TM^”, “VTV-PPV with Draeger Ventilator”, “T-Piece with RFM” and “T-Piece without RFM”. In all groups, mask PPV was provided with a size 0 round silicone face mask (Laerdal, Stavanger, Norway) to a modified infant manikin (ResusciBaby, Laerdal Medical Corp., Armonk, NY), fitted with a 30 mL test lung and made leak-free.^26^, ^27^ The compliance of the test lung is 0.5 mL/cmH_2_O. This manikin provided chest rise as feedback to the participants. Other clinical signs (heart rate, respiratory effort, SpO_2_) were provided verbally by a research assistant.

After providing written informed consent, participants had up to 10 min to familiarize themselves with the ventilation devices and respiratory function monitor. Randomization was revealed just prior to starting the first simulation. Participants then performed “VTV-PPV with NextStep^TM^”, “VTV-PPV with Draeger Ventilator”, “T-Piece with RFM” and “T-Piece without RFM” in the randomized order according to their allocation.

Four comparable scenarios involving a 27-week preterm infant, based on the RETAIN board game, were completed by each participant.^28^ To simulate a realistic resuscitation, participants were expected to perform the initial steps during which the baby was spontaneously breathing and was supported with continuous positive airway pressure (CPAP). Thirty seconds after starting CPAP, the infant became apneic and bradycardic (heart rate of 70–80/min) and participants were expected to initiate PPV. Participants then were expected to minimize mask leak by working through the first steps of MR.SOPA (M = adjust mask, R = reposition head, S = suction mouth and nose, O = open mouth, P = increase pressure). Participants could request a specific pressure increase with VTV-PPV with NextStep^TM^ or VTV-PPV with Draeger Ventilator and the person running the simulation would adjust pressure accordingly. When mask leak was either minimized, or when participants had completed the first five steps of MR.SOPA and too much time had elapsed without mask leak improvement, the heart rate rose to above 100/min and spontaneous breathing began. At that time participants were expected to switch back to CPAP for another 30sec before each scenario ended.

After completing all scenarios, participants completed a questionnaire about their experience with VTV-PPV with NextStep^TM^ using a Likert-scale (1 = Strongly Disagree, 2 = Disagree, 3 = Neutral, 4 = Agree, 5 = Strongly Agree).

### Data analysis

Respiratory data was only recorded and analysed during PPV (i.e., not during CPAP). For all respiratory variables, the median value for each recording was calculated, followed by the mean or median of those medians. Data were assessed for normality using the Shapiro-Wilk and Kolmogorov-Smirnov tests. Normality was met for positive end expiratory pressure, positive inflation pressure, and Ventilation Rate and analyzed using ANOVA for repeated measures using Bonferroni post-test. Because assumptions of normality were not met for V_T_ and mask leak, comparisons between groups were conducted using the Kruskal-Wallis rank test. Post hoc pairwise comparisons were performed using Dunn’s test with Bonferroni correction. The data are presented as mean (standard deviation-SD) for normally distributed variables and median (interquartile range-IQR) for skewed variables. All p-values are 2-sided, and p < 0.05 was considered statistically significant. All statistical analyses were performed using Stata 17 (StataCorp LLC, College Station, TX).

## Results

**Fig. 1:**
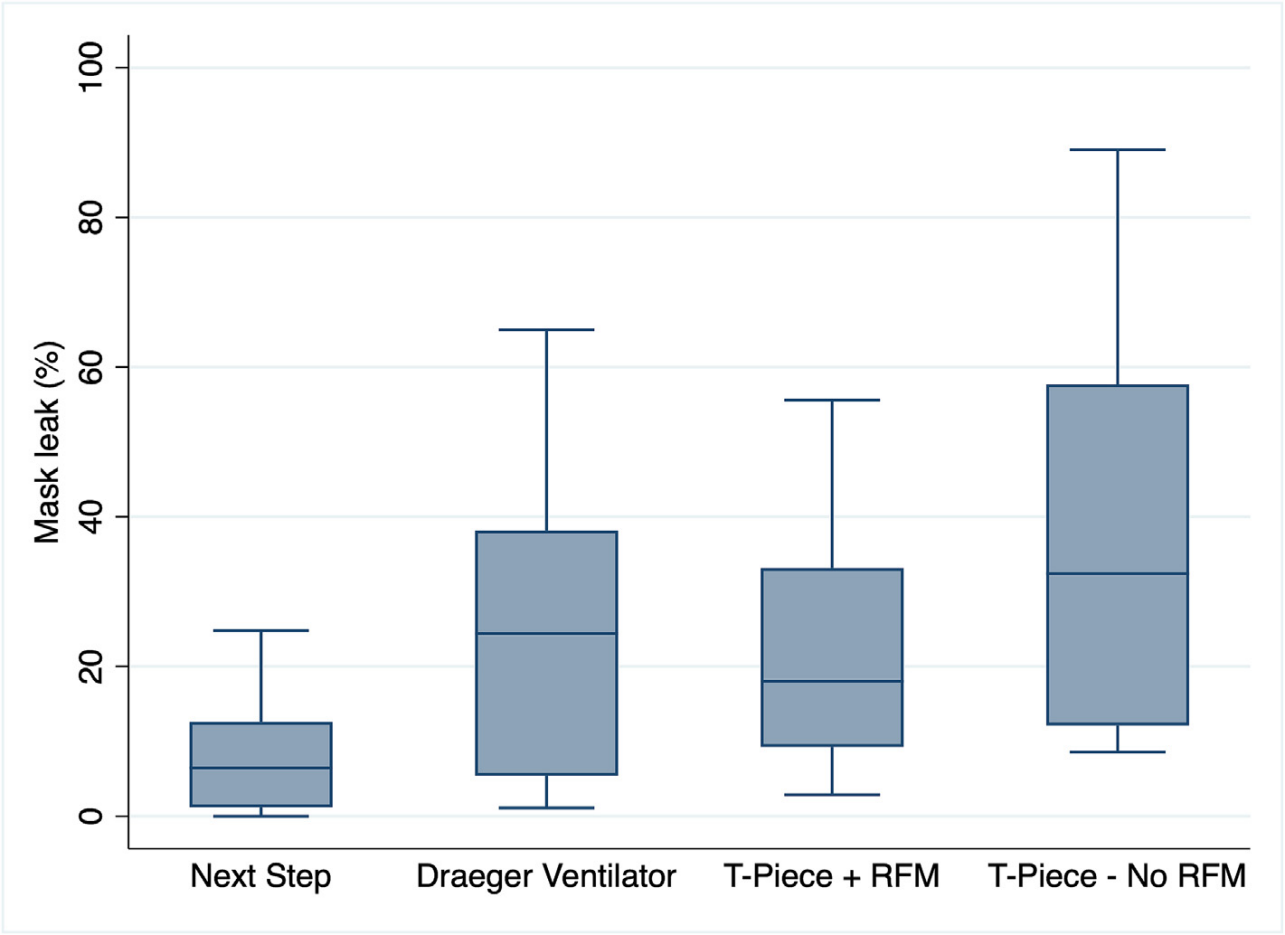
Mask leak (as % of expired tidal volume). Box plots show median (solid bar), interquartile range (margins of the box) and 95 % confidence interval. RFM = Respiratory Function Monitor.

**Fig. 2:**
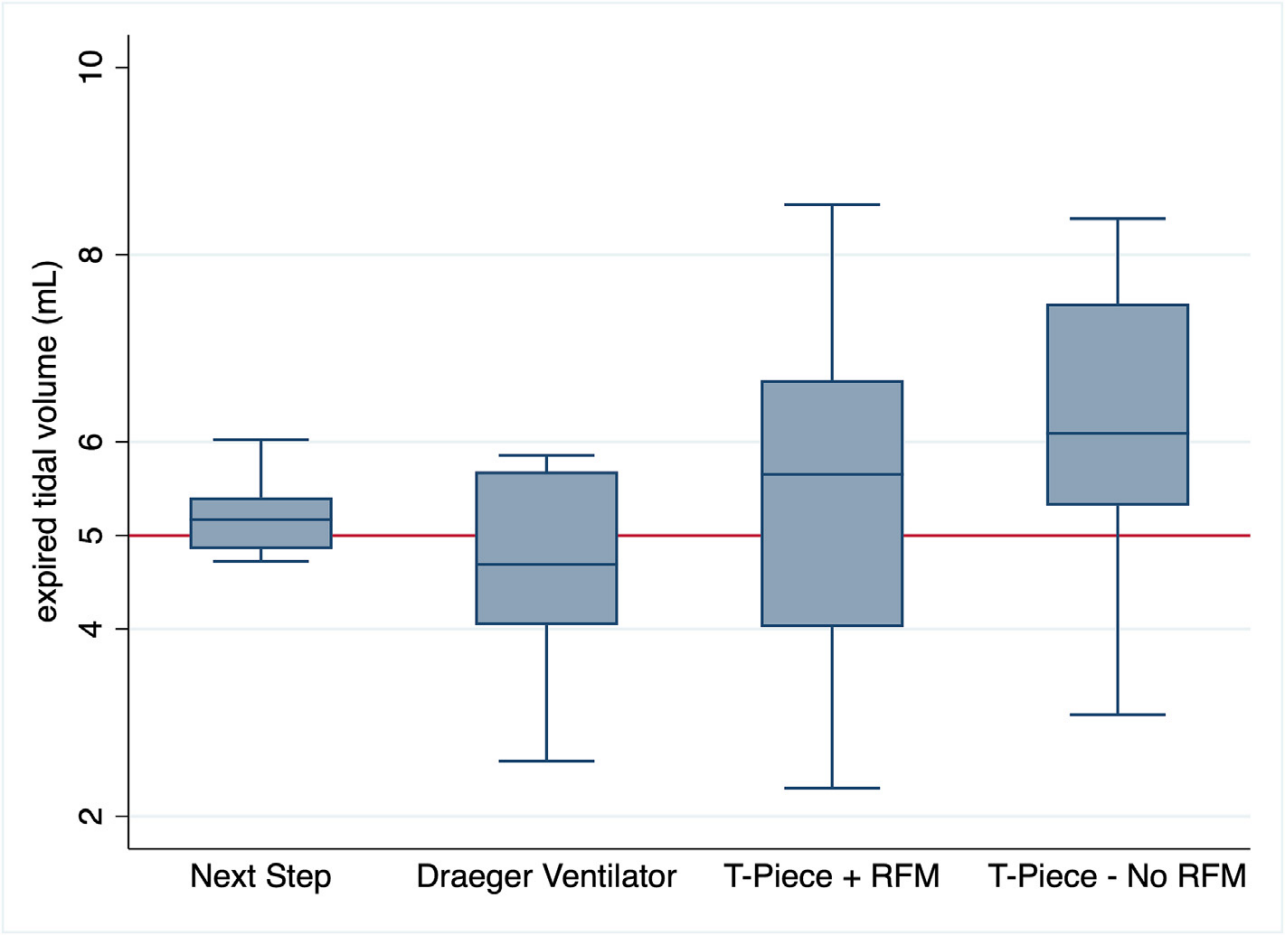
Expired tidal volume (mL/kg) with all four devices. Box plots show median (solid bar), interquartile range (margins of the box) and 95 % confidence interval. RFM = Respiratory Function Monitor.

Data collection occurred in July 2023. Among the 32 participants recruited, 7 (21.9 %) were male, and 25 (78.1 %) were female. The participants were comprised of 1 (3 %) neonatologists, 4 (13 %) neonatal subspecialty residents, 6 (18.5 %) neonatal nurse practitioners, 15 (47 %) respiratory therapists, and 6 (18.5 %) neonatal nurses. The participants had a mean (SD) of 10.6 (7.4) years of experience with neonatal resuscitation and had taken their most recent Neonatal Resuscitation Program course 11 (6.3) months prior to the study. All 32 participants performed were randomized to and completed one scenario using each of the four PPV devices. Respiratory variables are presented in Table 1 and Fig. 1 and Fig. 2. Overall, median (IQR) mask leak was significantly lower with VTV-PPV with NextStep^TM^ [6 (1–12)%] compared to the Draeger Ventilator [24 (25–38)%, p = 0.01], T-Piece with RFM [18 (9–33)%, p = 0.0088], and T-Piece without RFM [32 (12–57)%, p = 0.0001] (Table 1 and Fig. 1).

**Table 1:** Respiratory parameter.

**Variable**	**Next Step**	**Draeger Ventilator**	**T-Piece with RFM**	**T-Piece without RFM**
**Mask leak (%)**	6 (1–12)	24 (5–38)*	18 (9–33)*	32 (12–57)*
**Positive end expiratory pressure (mm Hg)**	5.0 (0.0)	6.6 (1.7)*	5.3 (0.7)	5.5 (0.8)
**Peak inflation pressure (mm Hg)**	17.2 (3.2)	16.5 (5.2)	24.0 (2.5)*#	24.6 (2.1)*#
**Ventilation rate (rate/min)**	50 (0)	50 (0)	40 (7)*#	39 (7)^*#^
**Tidal volume (mL)**	5.2 (4.9–5.4)	4.7 (4.1–5.8)	5.7 (4.0–6.7)	6.1 (5.3–7.5)

Data are presented as mean (SD).

* significantly different from the Next Step.

# significantly different from the Draeger Ventilator.

The median (IQR) V_T_ was 5.2 (4.9–5.4)mL with the NextStep^TM^, 4.7 (4.1–5.8)mL with Draeger Ventilator, 5.7 (4.0–6.7)mL with T-Piece with RFM, and 6.1 (5.3–7.5)mL with T-Piece without RFM (Table 1 and Fig. 2).

As each participant was exposed to all four interventions in a randomized order, the study followed a within-subjects repeated-measures crossover design. To minimize potential carryover or period effects, the order of interventions was randomized across participants. Visual inspection of the data and stratified analyses by treatment order did not indicate any systematic sequence or period-related trends, suggesting that carryover effects were unlikely to have influenced the results.

Post-simulation Survey Responses are presented in Table 2.

**Table 2:** Post-simulation survey responses.

**Question**	**Very Uncomfortable (1)**	**Uncomfortable (2)**	**Neutral (3)**	**Comfortable (4)**	**Very Comfortable (5)**
**Switching between CPAP and PPV with the Next Step**	1	5	5	9	12
**How do you feel not directly managing airway pressures with the Next Step during mask ventilation?**	3	4	9	11	5
**How do you feel about the MR. SOPA approach with the Next Step during mask ventilation?**	4	4	6	5	13

CPAP = Continuous Positive Airway Pressure, PPV = Positive Pressure Ventilation

## Discussion

To our knowledge, this is the first study to compare mask PPV during simulated neonatal resuscitation using VTV-PPV with the NextStep^TM^ versus a Draeger Ventilator or a T-piece with or without a visible RFM. With the NextStep^TM^, the i) mask leak was significantly reduced compared to all other devices (Fig. 1^,^ Table 1), ii) the delivered V_T_ was closer to the target of 5 mL/kg (Fig. 1^,^ Table 1), but not statistically significant, and iii) the peak inflation pressure was significantly lower than with T-Piece (Table 1).

A major limitation of mask-delivered PPV is the frequent occurrence of large and variable mask leak, even among experienced providers, which significantly compromises the delivered V_T_.^7^, ^9^, ^29^, ^30^ While the use of an RFM has been proposed to reduce mask leak, only two out of three randomized trials comparing visible versus masked RFM during delivery room PPV have demonstrated this benefit.^14^, ^15^ In our current study, mask leak was significantly reduced with the NextStep^TM^ compared to all other devices (Fig. 1, Table 1). It should be noted that participants used a two-hand mask technique during VTV-PPV, a method previously shown to significantly reduce mask leak.^31^ Therefore, it is possible that the observed reduction in mask leak was attributable to the ventilation technique rather than the mode of ventilation alone. However, the mask leak was significantly reduced with the NextStep^TM^ compared to the Draeger Ventilator, for which participants also used a two-hand technique. Though, a two-hand mask technique with T-Piece requires two providers, as a single provider requires one hand to provide ventilation leaving only one hand available to hold the mask.

Relying on manually set peak inflation pressure and subjective assessments of chest rise during mask PPV carries a risk of both under- and over-ventilation.^7^, ^9^, ^14^, ^15^, ^19^, ^29^, ^30^ This is particularly concerning given that delivery room studies have shown V_T_ during mask PPV can range widely − from 0 to 31 mL/kg.^7^ Such variability is alarming, as animal studies have demonstrated that lung and brain injury occur primarily due to excessive V_T_.^32^, ^33^, ^34^, ^35^, ^36^, ^37^, ^38^ These findings underscore the importance of precisely targeting delivered V_T_ during PPV. Studies incorporating RFMs have shown that visualizing the RFM can help reduce the incidence of excessive V_T_. This benefit has not been associated with a reduction in bronchopulmonary dysplasia, however it has been associated with a reduction in brain injury.^16^, ^17^ VTV-PPV may offer a more effective solution by providing tighter control of V_T_ than RFM use alone. Encouragingly, our study demonstrated that VTV-PPV with both the NextStep^TM^ and Draeger ventilator resulted in more consistent V_T_ targeting and required lower peak inflation pressure levels than with T-Piece. In our manikin test lung model, only a peak inflation pressure of 16 cmH_2_O was need… under optimal conditions, with constant lung compliance, to achieve a V_T_ of 5 mL/kg (Table 1), suggesting that effective V_T_ targeting and minimization of mask leak can significantly reduce the need for high peak inflation pressure. This combination of precise V_T_ control and lower peak inflation pressure suggests that VTV-PPV may reduce lung and brain injury from the onset of resuscitation. Moreover, in situations where lung aeration remains suboptimal, VTV-PPV offers an additional advantage: it can automatically increase pressure to achieve adequate aeration, thus avoiding delays linked to manual adjustments by healthcare professionals. At the same time, it maintains protective V_T_ targeting by capping peak inflation pressure at a set maximum and automatically reducing peak inflation pressure as lung aeration improves. This dual functionality may provide both immediate effectiveness and ongoing lung protection during the critical initial moments after birth. A concern with VTV-PVV with NextStep^TM^ is inadequate V_T_ delivery (<5 mL/kg) for low compliance (0.5 mL/cmH_2_O) lungs.^22^ Encouragingly, our study reported both adequate and consistent V_T_ delivery during VTV-PVV with NextStep^TM^ with a similar low compliance (0.5 mL/cmH_2_O) test lung.

Another potential advantage of VTV-PPV over T-Piece PPV is ability to provide of set and consistent ventilation rate. The 2020 American Heart Association Guidelines for Cardiopulmonary Resuscitation and Emergency Cardiovascular Care recommend a PPV rate of 40–60 breaths/min.^1^ PPV rate with T-Piece PPV without RFM was significantly lower than with VTV-PPV with the NextStep^TM^ (Table 1) and some participants provided PPV at a rate below the target 40–60 breaths/min with T-Piece. Although not yet studied, VTV-PPV may reduce the workload of timing ventilations as is required with T-Piece PPV.

Consistent with our previous studies evaluating healthcare professionals’ perceptions associated with VTV-PPV compared to the T-piece or Draeger Ventilator, most participants in the current study reported feeling comfortable with the technical aspects of VTV-PPV with the NextStep^TM^ (Table 2). These included transitioning between mask ventilation and CPAP, integrating VTV-PPV into corrective ventilation steps, and focusing on managing V_T_ rather than peak inflation pressure. If NextStep^TM^ were to be integrated into clinical practice, teaching and practice sessions would be required to ensure provider comfort with the device.

This study has several limitations. First, the participants were experienced healthcare professionals familiar with VTV using neonatal ventilators, which may limit the generalizability of the findings to less experienced users. Second, the manikin model employed in this study did not simulate the dynamic changes in lung compliance that occur after birth. Since VTV-PPV automatically adjusts peak inflation pressure in response to compliance changes, real-life conditions may further highlight its advantages. Additionally, the model did not account for spontaneous breathing efforts, airway secretions, or vocal cord movements, factors that can affect V_T_ through asynchrony or airway obstruction. These limitations underscore the need for clinical studies to validate our findings under real-world conditions.

## Conclusion

In a neonatal manikin model, VTV-PPV with the NextStep^TM^ using a two-handhold reduced mask leak compared to the T-piece without RFM guidance. Clinical studies to validate our findings under real-world conditions are warranted.

## Funding sources

There was no funding source for this study.

## CRediT authorship contribution statement

**Chelsea Morin:** Writing – review & editing, Methodology, Investigation, Formal analysis, Data curation, Conceptualization. **Kashmala Yousafzai:** Writing – original draft, Methodology, Investigation, Data curation, Conceptualization. **Brenda Hiu Yan Law:** . **Georg M. Schmölzer:** Writing – review & editing, Writing – original draft, Visualization, Validation, Supervision, Resources, Project administration, Methodology, Investigation, Formal analysis, Data curation, Conceptualization.

## Declaration of competing interest

None. The NextStep™ Neonatal Resuscitator (KM Medical, Auckland, New Zealand) was provided on loan by the manufacturer. No financial support was received for this study. The company had no role in the study design, data acquisition, data analysis, interpretation of results, or manuscript preparation.
